# Viewing biodiversity through the lens of science…and art!

**DOI:** 10.1186/s40064-016-2831-z

**Published:** 2016-07-26

**Authors:** David G. Angeler

**Affiliations:** Department of Aquatic Sciences and Assessment, Swedish University of Agricultural Sciences, Box 7050, 750 07 Uppsala, Sweden

**Keywords:** Art, Science, Ecology, Biodiversity, Beta diversity, Visuals, Pixels, Education, Sustainability, Statistics

## Abstract

With global environmental sustainability at the crossroads, approaches are needed to build an ecologically literate culture for collective societal navigation through the intricacies of swift environmental change. This paper demonstrates a transdisciplinary approach, grounded at the intersection between the arts and sciences, to increase awareness and understanding of the current biodiversity crisis. It focuses on one aspect of biodiversity, beta diversity, which examines how sets of animal and plant species differ between habitats. Theory and real examples of beta diversity of aquatic animal and plant species from dried-out ponds in Mediterranean Spain are presented in pixelized visuals. These visuals are artistic expression of and build the prior knowledge about beta diversity, which is scrutinized subsequently with statistical analyses to support the artistic approach with an objectively identified and numerically underpinned presentation of structure in the visuals. The choice to examine beta diversity in theory and reality first through art and then through science is deliberate. Combined, these aspects examine biodiversity through an eco-centric, rather than a species- and habitat centric view, incorporate elements of surprise (how can aquatic species in dry ecosystems survive), and reduce uncertainty (by providing a common numerical yardstick for interpreting the visuals). Together they can optimize a goal-directed learning process in the viewers necessary for making judgments, inducing affective reactions, and facilitating memory and decision making. The approach presented here provides an integral qualitative and quantitative model useful for a broader inductive-deductive education process towards finding sustainable solutions as our planet moves swiftly to a future without historical analogue. Combined art-sciences approaches, as the one presented here, are useful to facilitate citizens’ comprehension of the scientific and potential policy dimensions of environmental change, including biodiversity problems, especially because it is the general public that bears the costs of transformation and adaptation measures.

## Background

With global environmental sustainability at the crossroads (Kates et al. 2011), there is increasing recognition for the need of collaborative and transdisciplinary research efforts to address the challenges arising from a degrading planet (Scheffer et al. 2015). Collaborations between the arts (e.g., EcoArt) and sciences are mounting (e.g., Huws 2000; Neff et al. 2010), focusing on communicating the nature of problems, searching for new solutions, heightening awareness of ecological concerns, and designing ecological activity to enable public action (Wilson 2002; Kagan 2014). Visual communication of environmental issues has been particularly relevant to communicating the intricacies of the inherent complexity of environmental change across disciplines (Pink 2003; Nicholson-Cole 2005; Hansen and Machin 2013).

The approaches artists use to visualize environmental change problems range from realistic (e.g., photography, film, videos, TV) (Seppänen and Väliverronen 2003; Thomsen 2015) to abstract (Freeland 2004; Yusoff and Gabrys 2011), often aiming to elicit human tensions (beauty, ugliness; risk, security); for instance, by means of the toxic sublime (Peeples 2011). A widely recognized challenge in the arts is that subjectivity both in the artistic expression and the viewers’ emotional perception and reactions can lead to a decontextualization and a false understanding of patterns of order in nature. As a result, viewers may perceive sustainability problems as distant and unlinked to personal experience and exposure (Lorenzoni et al. 2007; Moser 2010).

Scientific disciplines dealing with sustainability challenges such as ecology and the environmental sciences aim at presenting environmental problems (e.g., climate change) in the form of results from often complex statistical and modeling approaches. However, many of the visualizations based on models (e.g., climate maps) or output formats for numbers (graphs) only represent the results of a highly complex, deductive and artificial process. Abstraction and artificiality in scientific visualizations, while representing the order of nature and environmental change problems objectively, can also remove the viewer from context. Similar to the artistic approach, scientific visualizations of environmental problems can lead to a disconnect between peoples emotions to and experiences from nature (Schneider 2012).

This paper demonstrates how an ecological concept, biodiversity, which has gained center stage in the environmental sustainability debate (Novacek 2008), can be expressed artistically and made accessible to the viewer. Artistic expressions of biodiversity are deemed relevant to increase the public awareness about the current unprecedented rates of human-induced species extinctions (Bellard et al. 2012). This paper examines specifically one aspect of biodiversity: beta diversity. Rather than studying how many species of plants and animals can be present in a habitat, beta diversity quantifies how the sets of species differ among sites in a specific region (Whittaker 1960). Beta diversity, which scientist express numerically, has been valuable for studying environmental change problems; that is, how urban expansion, deforestation, increasing agriculture and other forms of landuse change have contributed to a faunal and floral homogenization across habitats in a region (e.g., Olden et al. 2006; Gámez-Virués et al. 2015). More similar faunas and floras across sites can be seen, for example, in the form of extinction of emblematic species such as charismatic, valued vertebrates or taxa of socioeconomic interests (e.g., fish game species) in selected sites or an increase of different pest or exotic species across habitats.

In the context of beta diversity, crucial theoretical building blocks are nestedness, which emphasizes species extinctions across sites (e.g., the loss of valued game and fish in the above example) and turnover, which focuses on how the sets of species change across sites (exemplified by pest species above) (Baselga 2012). In theory, nestedness or turnover take a basic expression in the form of a perfect structure, but these perfect representations do not exist in nature. There is a plethora of studies in terrestrial and aquatic environments that show that it is always a mixture of both. An obvious dilemma arises that has intrigued philosophers incessantly: perfect nestedness and turnover are unobservable; they cannot be deduced scientifically from observations of nature. As heuristics existing in theory, they provide the inductive basis for seeking scientific representation of knowledge. This further complicates the laypeople’s understanding of ecological concepts necessary for comprehending sustainability problems. Without training in science, laypeople perceive theory as conforming to the monolithic logic and perception of science associated with rationalization. This further increases the risk of impoverished views and understanding of scientific representations of nature, and especially sustainability challenges (Locke 2001). Ultimately, this may reinforce peoples emotional disconnect from environmental problems.

This paper aims at reconciling artistic and scientific approaches in an attempt to communicate biodiversity in general, and beta diversity in particular to the public. Using pixel art to visualize species and ecosystems in the form of simple geometric shapes, this paper will present the theoretical constructs (perfect nestedness and turnover) of beta diversity, and the beta diversity of real assemblages of animals and plants from dried-out pond ecosystems, in a semiarid environment where the impacts of climate change are substantial (Gibelin and Déqué 2003). These visuals as artistic expression of beta diversity are subsequently scrutinized with statistical analyses to support the artistic approach with an objectively identified and numerically underpinned presentation of structure in the visuals. The combined art-science approach presented here shall offer several benefits for environmental education: (1) Focusing on biodiversity through an eco-centric, rather than a species- and habitat centric view, and by examining beta diversity as one aspect of biodiversity. (2) Provide opportunities to make abstract ecological theory tangible to laypeople. Combined with the real examples, envisioning of the broader hypothetical-deductive process, which is considered important for engaging the public with learning and understanding of environmental problems, can be improved (Miller 2001). (3) Incorporate elements of surprise by purposefully choosing habitats from a semiarid environment that, despite being dry, still harbor a wealth of animal and plant diversity. (4) Reduce uncertainty in learning by providing a common numerical yardstick for interpreting the visuals. These aspects combined shall facilitate the goal-directed nature of information processing that are necessary for making social judgments, inducing affective reactions, and facilitating memory and behavioral decision making (Wyer and Srull 1986).

## Methods

### Data for real examples

The real examples for this study were purposefully chosen to maximize the surprise component to boost the learning process (Lisman and Grace 2005). The examples build on dried-out pond ecosystems that are apparently lifeless, but which harbor a great amount of biodiversity in the form of seeds and resting stages in the dry sediments. That is, there is a latent biodiversity that manifests itself once the ponds refill with water. Many of the animal and plant species that emerge from these dry sediments are microscopic and thus not visible to the human eye. Visualizing this “unseen” diversity can further reinforce the surprise element for the viewer.

**Fig. 1:**
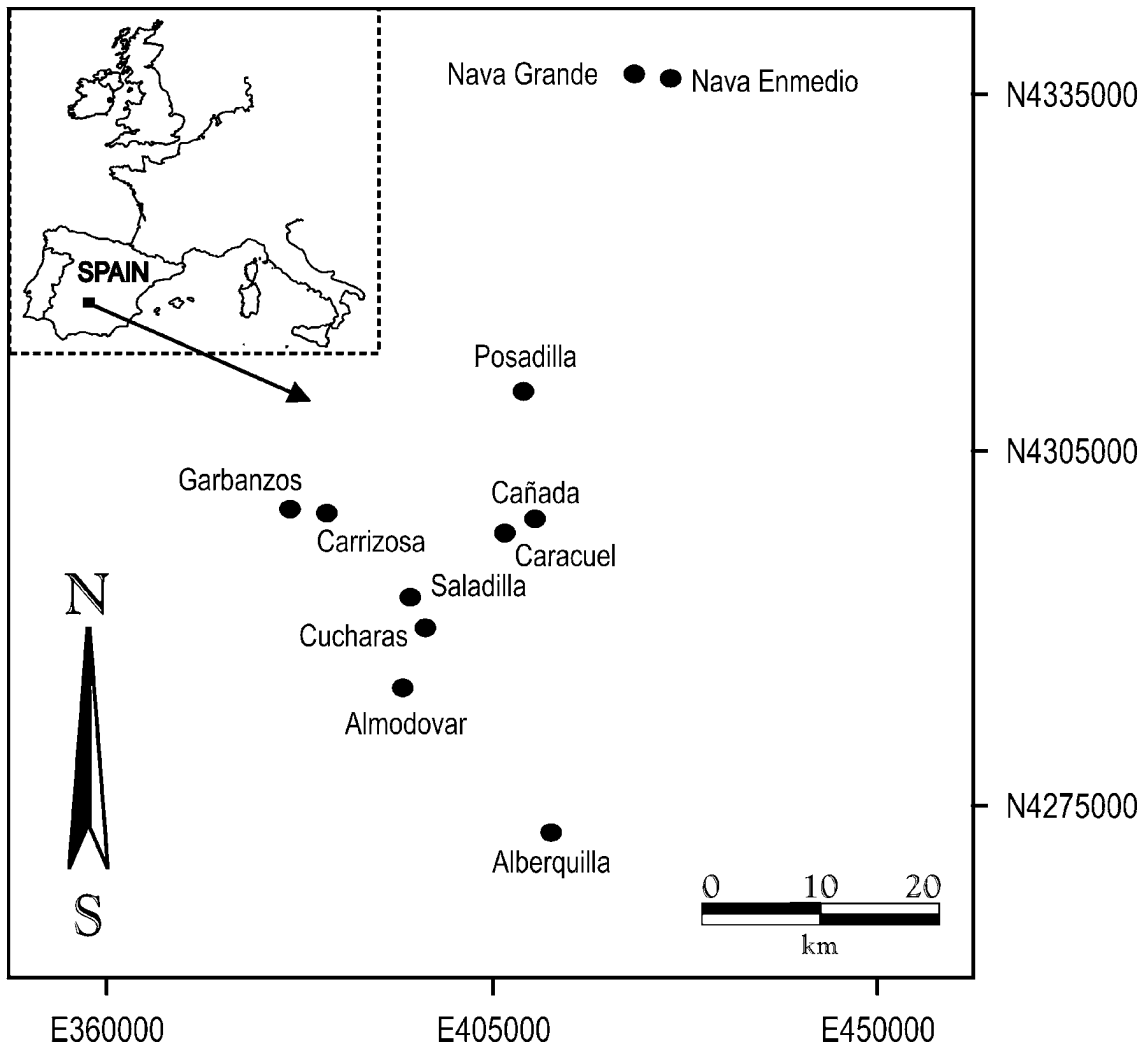
Location of study sites in the Campo de Calatrava area (central Spain). Geographical coordinates are given as UTM positions (north and east coordinates in zone 30)

The data used for representing beta diversity artistically were obtained from The Campo de Calatrava area in central Spain (39**°**00**′**N, 4**°**25**′**W; 38**°**30**′**N, 3**°**23**′**W), which covers 12 227 ha. Despite historical degradation by agriculture practices, the area contains a unique wetland fauna and flora, highlighting the value of the remaining wetlands for regional biodiversity (Velayos et al. 1989; Alonso 1996; Sánchez and Angeler 2007), and which can be visualized artistically. For the present study, eleven wetlands were chosen that represented abiotic variability, landscape characteristics and anthropogenic stress conditions among wetlands in the region (García-Canseco 2000) (Table 1; Fig. 1). In 2005, sediments were collected in these ponds when they were dried out as a result of a prolonged drought event. These sediments harbor a significant amount of dormant propagules and seeds of animals and plants. The sediments were rewetted in plastic containers, inducing experimental hatching of these resting stages. The plants and animals hatched from these sediments were sampled and taxonomically identified following the protocol by Angeler et al. (2008). A list of these species is presented in Appendix 1.

**Table 1:** Overview of ponds sampled for invertebrates and macrophytes

Wetland	Geographic location (UTM coordinates)	Municipality	Altitude (m)	Wetland size (ha)	Distance to nearest pond (km)	Geomorphic type
Alberquilla	Z30, E410731, N4272899	Mestanza	833	6.5	16.98	Crater
Almodovar	Z30, E398387, N4285646	Almodóvar de Calatrava	640	22.6	5.49	Crater
Cañada	Z30, E409333, N4298926	Cañada de Calatrava	607	60.2	2.81	Piedmont
Caracuel	Z30, E407498, N4297226	Caracuel de Calatrava	627	61.4	2.82	Piedmont
Carrizosa	Z30, E392261, N4299942	Cabezarados	654	21.1	3.24	Piedmont
Cucharas	Z30, E399958, N4289407	Villamayor de Calatrava	609	106.8	2.95	Depression
Garbanzos	Z30, E389009, N4299957	Cabezarados	638	21.6	3.24	Depression
Nava Enmedio	Z30, E4214444, N4336323	Malagón	586	36.2	2.63	Piedmont
Nava Grande	Z30, E418069, N4337275	Malagón	578	110.2	2.63	Piedmont
Posadilla	Z30, E408938, N4310859	Ciudad Real	633	13.4	19.05	Crater
Saladilla	Z30, E399913, N4292226	Villamayor de Calatrava	609	39.3	2.95	Depression

### Matrix construction for analysis and visual representation

**Fig. 2:**
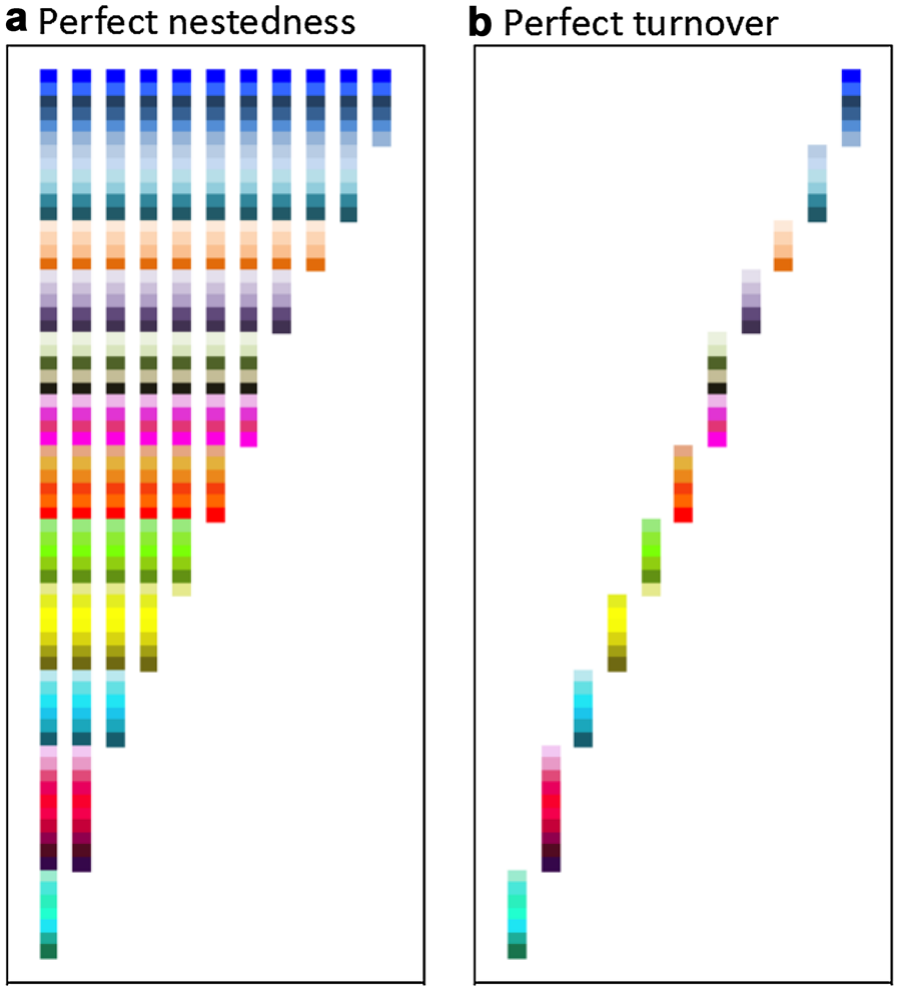
Pixel visuals showing perfect nestedness (**a**), turnover (**b**). *Each column* presents a hypothetical habitat/ecosystems (n = 11), and *each color pixel* represents a hypothetical species

**Fig. 3:**
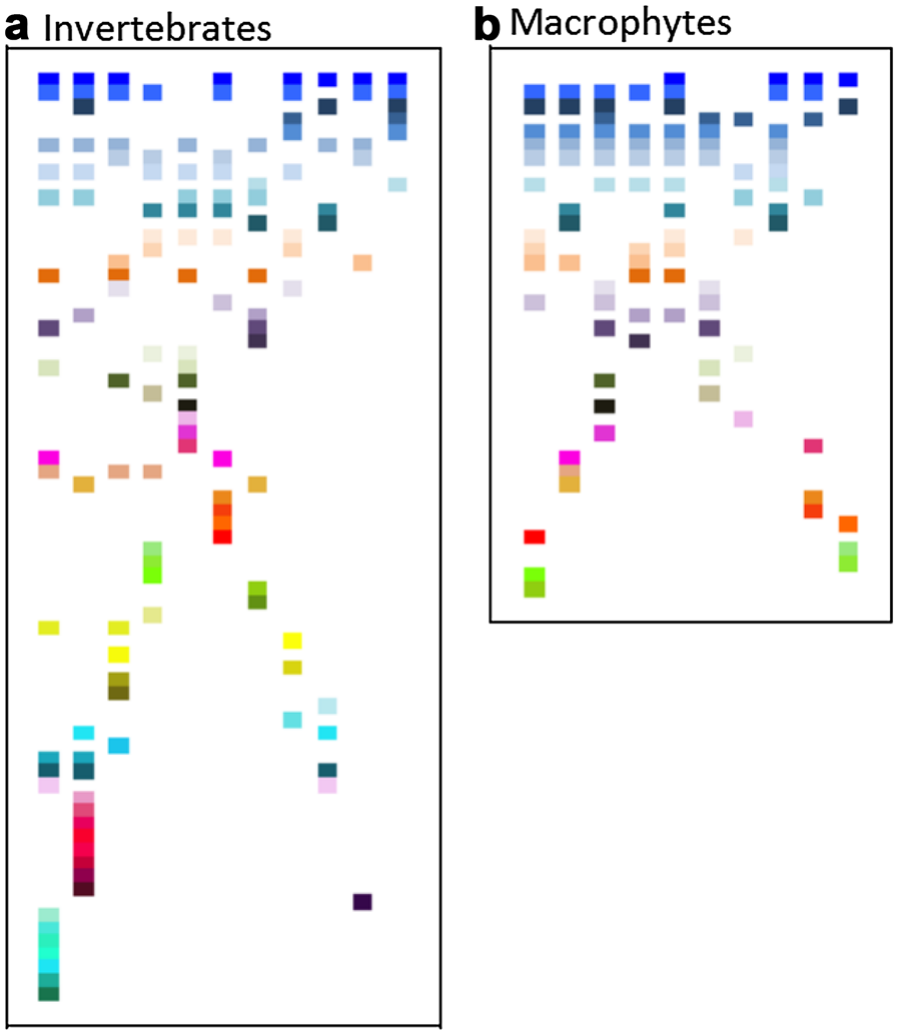
Real examples of beta diversity represented through pixel art; **a** invertebrates, **b** macrophytes in a pond complex (n = 11 for invertebrates; n = 10 for macrophytes due to the lack of species in one pond) in Spain

The approach to express beta diversity artistically is based on pixelation, allowing for a simple expression of sets of species within and across the eleven ponds, whereby every species comprises a pixel. The pixels are stacked in columns, which represent the ponds. The representation of the perfect fractions of beta diversity (nestedness and turnover) follows the same approach. The resulting visuals take specific geometric shapes that make the scientific information tangible (Figs. 2, 3). The use of pixel elements in this study builds on visual expressions used in different modern and contemporary art movements, including, for instance, color field, Bauhaus, De Stijl, New Media art, and VJing. Also, pixels comprise the basic building blocks that characterize the video gaming art movement, a specialized form of computer art employing video games as the artistic medium (Parker 2013).

Matrices for the visualization and statistical analyses of real examples and pure nestedness and turnover were constructed in Microsoft^®^ Excel^®^ for MAC v. 14.4.7. Each square in the matrices (pixel) was 1 × 1 cm and symbolized a species. The habitats are represented as columns. To better discern between habitats in the visuals blank columns were included. The real invertebrate examples contained 11 habitats/ponds (columns) and a total of 1875 pixels to represent the species across ponds in these visuals. The pond with the highest richness contained 21 species. The real macrophyte example contained less species (pond with highest richness, n = 13) and only 10 habitats due to the lack of plant occurrence in one pond compared to the invertebrate example; therefore, its visual expression and data analysis is based on a smaller matrix (1012 pixels). The matrices of the perfect representation of nestedness and turnover were sized to fit the real example of invertebrates (1875 pixels); that is, there were 11 columns expressing hypothetical habitats and species richness as in the real example of invertebrates.

### Statistical analyses of visuals

To inform the viewer about how nestedness and turnover are related in expressing beta diversity, I first examined how much these fractions explain numerically beta diversity in the visuals. The statistical approach is based on Baselga and Orme (2012). It first calculates an overall metric of beta diversity, which allows assessing the degree of compositional differences in the set of species across sites; i.e., whether or not sites share many species. Next, the algorithm partitions the beta diversity metric into its nestedness and turnover components, allowing to assess which fraction dominates in the real examples. Details about this approach are summarized in Appendix 2.

In the next analysis I assessed the structure that is present in the geometric shapes of the visuals derived from each matrix (perfect nestedness, perfect turnover, invertebrates, macrophytes). This analysis aimed at reducing uncertainty in the interpretation of the visuals through an objective identification of structure that can be expressed numerically in a simple way. I used a spatial modeling technique commonly applied in ecology (Dray et al. 2006). A detailed description of each step in the modeling is given in Appendix 2. In short, the modeling can identify hierarchical and orthogonal (statistically independent) patterns present in paintings and other visual artistic expressions. Revealing these hierarchical and/or orthogonal structures provides insight into the complexity and order inherent in visual art. Moreover, the modeling allows for a numerical quantification of this structural complexity, using the amount of adjusted variance explained, a metric of model performance. That is, it permits discerning objectively patterns (spatial structure or order present) from noise (absent structure) in the geometrical structures of art works; i.e., a lower variance explained indicates a higher degree of noise relative to order and vice versa. This modeling approach is thus a valuable scientific tool for objectively analyzing structure in visual art works; it overcomes bias that can arise based on the subjective interpretations. Details about the approach are given in Appendix 2.

## Results

The perfect nature of nestedness and turnover were expressed artistically using pixel images (Fig. 2). In this artistic expression the color columns represent different hypothetical habitats or ecosystems. Within each column the pixels of different colors express the sets of hypothetical species present within each ecosystem. In the perfect nestedness example there is a gradual decrease in the sets of pixels/species from left to right, highlighting the nested structure of habitats and the associated extinction of species. The perfect turnover example shows how the set of squares changes between the columns; no set of species overlaps between habitats. A high degree of spatial geometry characterizes nestedness and turnover in their perfect expression. The former takes a triangular and the latter a diagonal shape (Fig. 2).

Figure 3 shows beta diversity in real examples, based on the mixture between turnover and nestedness components in invertebrate and plant assemblages in the pond complex. These are expressed artistically in the same way as perfect nestedness and turnover. Examining the distribution of species (pixels) within ponds (columns) reveals a clear break up of geometry in the artistic expression of beta diversity in these real examples, relative to perfect representations. The emergent structure resulting from the symmetry break up during the structural “metamorphosis” from theory to reality becomes reminiscent of stickmen (the authors own subjective interpretation).

The statistical approach to quantify beta diversity shows overall high beta diversity in the visuals of real examples (Table 2): 0.87, invertebrates; 0.84 macrophytes [on a scale between 0 (lowest) to 1 (highest)], indicating a high degree of compositional distinctness in the sets of species across the ponds. In both visuals of organism groups the break of symmetry could be attributed to a dominance of turnover (0.84, invertebrates; 0.79, macrophytes), relative to nestedness (0.03, invertebrates; 0.05, macrophytes) (Table 2). In the visual of the perfect turnover, beta diversity has a value of 1 (no shared species across habitats). When partitioned statistically, turnover logically becomes 1 and nestedness 0. In the visual of perfect nestedness beta diversity has a value of 0, and it partitions into equal parts (0.68 for nestedness and turnover) (Table 2). Methodologically, this indicates that beta diversity in its mathematical expression is entirely explained by nestedness (A. Baselga, personal communication).

**Table 2:** Results from the beta diversity partitioning analyses that assessed the relative importance of nestedness and turnover components in the visuals

Analysis/fraction	Invertebrates	Plants	Perfect turnover	Perfect nestedness
*Partitioning analysis*				
Overall beta diversity	0.87	0.84	1	0
Turnover	0.84	0.79	1	0.68
Nestedness	0.03	0.05	0	0.68
*Modeling analysis*				
Structure	0.26	0.14	0.80	0.84
Noise	0.74	0.86	0.20	0.16

Also shown art the results from the spatial modeling analyses that assessed the amount of structure versus noise in the visuals. Numbers show the amount of variation explained

The second statistical exploration that quantified the degree of structure present in the geometric shapes revealed high structure in the visuals of perfect turnover and nestedness [explanatory power of the minimum model (i.e., the adjusted variance explained): turnover, 0.80; macrophytes, 0.84] (Table 2). Noise, which comprised the difference of these values to 1 (the highest possible variance), was comparatively low (0.20, invertebrates; 0.16 macrophytes) (Table 2). These values changed drastically in the real examples, coinciding with the break up of geometry observed in the visuals: invertebrates, 0.26 structure/0.74 noise; macrophytes, 0.14 structure/0.86 noise (Table 2). This analysis also revealed that the patterns of order were structurally simple. That is, no orthogonal or hierarchical patterns indicating complexity of orderly structure were present in the visuals.

## Discussion

The environmental challenges ahead are complex and multidimensional (Angeler et al. 2016a, b). Scientists, artists and educators play an important role in bringing about an ecologically literate culture necessary for environmentally responsibility (Hicks and King 2007). This paper aimed at reconciling artistic and scientific approaches to engage people with biodiversity issues. Visuals based on simple pixelized geometric shapes were used to demonstrate an approach that could elicit emotional reactions in people, increase awareness, facilitate learning and understanding, and ultimately critical thinking about biodiversity with an improved knowledge grounded in the ecological sciences. The approach follows Thomsen (2015) that reconceptualizes “seeing” as “questioning”, rather than believing. In this process a chain of questioning—for instance, what is biodiversity and why the need to study it? What is beta diversity and how does it relate to biodiversity? What are elements of uncertainty and surprise, and how do they manifest?—can improve the learning process and connect people more closely with biodiversity and sustainability issues (e.g., Ryan 2001).

Recognizing that current biodiversity loss resembles a 6th mass extinction in Earth’s history (Bellard et al. 2012), there is critical need to engage people with biodiversity issues (Novacek 2008). Given that with the publication of the first landmark book “The Diversity of Life” (Wilson 1992) biodiversity became the subject of school and academic courses, public journalism, television specials, and major museum exhibits, most people may have acquired basic understanding of the meaning of the word biodiversity (Novacek 2008); however, specifics of biodiversity may go unnoticed. In ecology, biodiversity is a portmanteau that encompasses many different meanings and facets of biological diversity (Magurran 2003). It includes not only the richness of plant and animal species within and across habitats and entire regions, but also variation in their abundances, their genetic diversity and variability of functional traits. These traits allow them carry out important processes like production of food and timber, decomposition of dead material, pollution and erosion control, to list a few, and these provide important ecological, aesthetic and economic values for humans (Truchy et al. 2015). This multifaceted character of biodiversity may be only known to a limited number of scientists (ecologists and environmental scientists). Scientists working in other fields (e.g., technology, economy, politics), let alone the broader public without scientific training, may be unaware of this varied meanings of biodiversity. This unawareness, together with the media and public prioritizing other problems (economy, health, terrorism) than biodiversity loss, results in a failure to recognize the implications of biodiversity issues in exacerbating many problems more familiar and more important to people (Novacek 2008).

Seppänen and Väliverronen (2003) advocated using case-by-case studies to allow the viewer deduce causes, consequences and potential remedies of environmental change. It is clear that the presentation of biodiversity can benefit from such an approach, given that the multiple meanings and components of biodiversity may overwhelm people and reinforce a decontextualization and disconnect of them with these issues. In this paper, beta diversity has been chosen as one aspect of biodiversity, which specifically assesses the difference in assemblage structure of plant and animals between habitats in a region. As is the case with the broader concept of biodiversity, beta diversity has become an umbrella term in the ecological sciences, mainly because of the different approaches ecologists have developed for quantifying compositional heterogeneity between habitats (Tuomisto 2010). Many of these approaches not only allow for analyzing species presence-absences across sites, but also explicitly account for the abundances of these species for research questions where community evenness is relevant (Anderson et al. 2011). The use of beta diversity as an umbrella concept is deemed suitable for communicating the concept because the specifics of scientific quantification approaches are irrelevant for the purpose to increase laypeople’s awareness and knowledge about its meaning. For simplicity, and to facilitate the layman’s learning process, this study has focused on how the sets of species vary across sites.

The choice of beta diversity is grounded in the fact that public environmental discourse and funding for biodiversity conservation often focuses on single species, such as charismatic megafauna (e.g., elephants, pandas, tigers), and specific habitat (rainforests) protection (Vandermeer and Perfecto 1995; Bowen-Jones and Entwistle 2002). However, species and habitat-centered views may cause substantial misunderstanding of extinction processes, and ultimately suboptimal biodiversity management because the broader interacting ecological factors (abiotic and biotic) are ignored (Hunter and Brehm 2003; Failing and Gregory 2003). For instance, by focusing only on charismatic species the role of smaller organisms that are the most specialized and the most vulnerable to extinction from human disruption are ignored (Vandermeer and Perfecto 1995).

Beta diversity focuses the meaning on regional biodiversity from an eco-centric, rather than a species- or habitat centric view, allowing people to envision and understand spatial aspects of extinctions. The visual of perfect nestedness, despite being a theoretical construct that cannot be seen in nature, has the educational value of communicating to people the ecological phenomenon of species extinctions in a spatially implicit approach. The way the loss of pixels (or species) between adjacent ecosystems (columns) is arranged comprises one among multiple ways to demonstrate and communicate extinctions associated with beta diversity (i.e., loss of species across sites). Notwithstanding, the use of pixels and their purposeful arrangement in the visuals allows giving equal weight to species. This presentation achieves neutrality regarding the perceived socio-economic value of taxa by people. That is, by down-emphasizing value-laden connotations of animals and plants, thinking about biodiversity from an eco-centric rather than species-centered perspective can be spurred.

Presenting turnover, another aspect of beta diversity, concomitantly to extinctions, further facilitates the presentation of beta diversity as an eco-centric concept. It essentially shows that other processes than only extinctions are relevant for envisioning and understanding biodiversity. The change of the sets of pixels between habitats in the visual of perfect turnover differs drastically from that of perfect nestedness, leading to the emergence of geometric shapes, a triangle (nestedness) and diagonal (turnover) respectively, that allows distinguishing both concepts instantaneously without the need of previous knowledge in the viewer. These distinct shapes provide a rapid means to demonstrate, communicate and let the viewer assimilate the multiple facets of biodiversity at large, and beta diversity in particular.

There is also added value to the visual presentations of the perfect patterns of extinctions and turnover. Visuals provide opportunities to make abstract ecological theory tangible to laypeople and potentially contribute to break with their notions that theory conforms to a monolithic logic and perception of science associated with rationalization (Locke 2001). Furthermore, the clear geometries of shapes associated with these theoretical constructs has potential to induce critical thinking not only about biodiversity per se, but about the order and structure of nature in general. Contrasted with the visuals of real animal and plant communities, the viewer can contemplate how order changes between theory and empiricism. The “metamorphosis” of shapes from theory to reality has potential to let the viewer benchmark the realistically observed order in nature against those that would exist in a hypothetically perfectly structured but unrealistic world. This metamorphosis becomes allegorical to the broader hypothetical-deductive process, the *raison d’être* of scientific endeavor, which is considered important for engaging the public with learning and understanding of environmental problems (Miller 2001).

The purpose of contemplating such a metamorphosis can also be scrutinized from an uncertainty viewpoint. Uncertainty is arguably a major obstacle for comprehending considerable barriers to the acceptance and understanding of environmental problems (Thomsen 2015). At first glance, the visuals of beta diversity may not be exempt from such uncertainty. The transforming geometries resulting from the metamorphosing shapes turned from clear structure embodied in perfect turnover and nestedness to forms that have been interpreted as stickman in the real examples of animals and plants in ponds. The reference to the author’s own subjective interpretation of the real examples is deliberate. It shall demonstrate that the loss of clarity of patterns of the visuals during the metamorphosis opens up for the possibility of multiple competing associations in viewers contemplating these visuals. Different ways of individual interpretation increases a deductive uncertainty and therefore ambiguity among people collectively in comprehending and envisioning ecological phenomena, exemplified by beta diversity here. While this subjectivity may hardly be entirely overcome, this study uses the resulting uncertainty as an opportunity to build common ground for further examination and comprehension by the viewer by means of a subsequent statistical analysis. In this context, uncertainty can be used to stimulate public reactions to and discourses with environmental issues (Hansen 2010).

In this study, uncertainty arising from visual examination is considered to build the prior knowledge upon which further learning can be constructed. Statistical analysis was used to integrate the prior knowledge obtained from the visualization of beta diversity with aspects of the learning process. Specifically, it can help the goal-directed nature of information processing necessary for making judgments, inducing affective reactions, and facilitating memory formation and decision making (Wyer and Srull 1986). The statistical analysis sets a common interpretational yardstick for viewers by presenting objectively identified patterns of structure in the visuals that is expressed objectively through numbers from the modeling. The approach allows deducing why structure is higher in one visual (0.26 of variance explained in visuals of animals) relative to the other (0.14 in visual of plants), and why structure in these real examples is generally low. There is a wide range of implications following from this for envisioning biodiversity that deserve closer scrutiny but this is beyond the scope of this study which targets uncertainty. The advantage of numbers infallibly reflecting structure and order in the visuals can address, and potentially partly overcome, subjective interpretation obtained from prior knowledge deduced from the examination of visuals. It can contribute to homogenize a subsequent thought processes across viewers that eventually reduces uncertainty (Poole 2009). Reducing uncertainty is a major goal for navigating through the complexities of environmental change affecting many of the intricacies of sustainability (Berkes et al. 2008). Reduced uncertainty in public understanding of biodiversity should be one way towards comprehending these complexities.

A further advantage of underpinning visual expressions of beta diversity with statistical analyses is the possibility to bolster learning by including elements of curiosity and surprise. In the example of perfect turnover, the partitioning of the entire variance to this component (that is, 1 for turnover, 0 for nestedness) seems logic. However, in the perfect nestedness example the partitioning of the variance in equal fractions may be a priori a surprise for most viewers without knowledge in mathematical calculus. This element of surprise, achieved through a scientific approach, has great value in environmental communication because surprise and novelty stimulate learning and long-term memory formation (Lisman and Grace 2005).

To further bolster the surprise effect the examples of plants and animal communities in ponds in a semiarid climate have been chosen on purpose. The impacts of climate change in dryland countries may be substantial (Alvarez-Cobelas et al. 2005). The duration, frequency and magnitude of droughts is increasing, threatening water resources, and boosting a desertification process (Reynolds et al. 2007). Ultimately this can augment the biodiversity crisis in such areas. People experience and emotionally react to heat waves, failing crops, dying cattle and water shortages for human consumption and agricultural irrigation with a sense of desperation (Thomas et al. 2007). In fact, the ponds used in this study were dry when sampled during a prolonged drought period in Spain. The dry state of ponds for several years, evident in cracked soils and lack of life, potentially reinforces people’s negative emotions through a sense of doom. However, dry and apparent lifeless ponds store a wealth of seeds, eggs and other propagules that give birth to new life, ranging from microscopic to macroscopic organisms, once harsh environmental periods are overcome; that is, when the ponds refill after drought. Therein consists the surprise element. It may foster peoples learning about the resilience of nature, and the ecological strategies of organisms and ecosystems to buffer against harsh environmental conditions. The bottom line is that the choice of examples, like ponds in a dryland country used in this study, can be used to maximally exploit the surprise element in education about biodiversity and other environmental aspects.

## Conclusions

Since the costs of both prevention of and adaptation to environmental change, including biodiversity issues, must be borne by the general public, the comprehension of the scientific and policy issues by citizens is crucial to political decision making (Kempton 1991). This paper shows how a combined art-science approach can serve as an education tool for the public, by letting the viewers engage emotionally with complex ecological theory and its application to environmental studies. The example showcased here highlight this potential from a potentially vast spectrum of application to communicate environmental sustainability challenges in general and biodiversity in particular. It provides an integral qualitative and quantitative model useful for a broader inductive-deductive learning process. It expands on qualitative approaches in transdisciplinary arts and sciences research (Parkinson 2008; Österblom et al. 2015; Angeler et al. 2016a, b) striving for finding sustainable solutions as our planet moves swiftly towards a future without historical analogue.

## Appendix 1: List of invertebrate and plant species with authorities occurring in the ponds

**Table:**

Invertebrates	Plants
*Acartia* cf*. clausi*	*Agrostis pourretii* Wildenow
*Acroperus neglectus* Liljeborg	*Alopecurus* sp.
*Alona qaudrangularis* Müller	*Anthemis cotula* Linné
*Alona guttata* Sars	*Baldellia ranunculoides* (Linné) Parl.
*Alona* sp.	*Chara connivens* Salzm. ex A. Braun.
*Alonella excise* Fischer	*Chara galioides* De Candolle
*Biapertura affinis* Leydig	*Chenopodium* sp.
*Branchinecta ferox* Milne-Edwards	*Crypsis aculeata* (Linné) Aiton
*Branchinecta orientalis* Sars	*Damasonium polyspermum* Coss.
*Candonopsis* sp.	*Elatine macropoda* Gussone
*Ceriodaphnia dubia* Richard	*Frankenia pulverulenta* Linné
*Ceriodaphnia reticulata* Jurine	*Hordeum marinum* Huds.
*Chydorus latus* Sars	*Isolepis setacea* (Linné) R. Br.
*Chydorus piger* Sars	*Juncus articulatus* Linné
*Chydorus sphaericus* Leach	*Juncus buffonius* Linné
*Conchostraca unidentified species*	*Juncus gerardii* Loisel
*Cyclocypris* sp.	*Juncus pygmaeus* Rich.
*Cypria* sp.	*Lolium rigidum* Gaud.
*Daphnia cucullata* Sars	*Lotus* sp.
*Daphnia curvirostris* Eylman	*Lythrum flexuosum* Lag.
*Daphnia longispina* Müller	*Lythrum hyssopifolia* Linné
*Daphnia similis* Claus	*Myriophyllum alterniflorum* De Candolle
*Darwinula* sp.	*Plantago coronopus* Linné
*Darwinula stevensoni* Brady & Roberston	*Poa bulbosa* Linné
*Diacyclops* sp.	*Polygonum bellardii* All.
*Diaphanosoma brachyurum* Liévin	*Polypogon maritimus* Willdenow
*Diaphanosoma mongolianum* Uéno	*Polypogon monspeliensis* (Linné) Desf.
*Diaptomus* sp.	*Pueccinellia* cf. *fasciculata* (Torrey) E.P. Bicknell
*Dunhevedia crassa* King	*Pulicaria paludosa* Link.
*Ectocyclops phaleratus* Koch	*Ranunculus peltatus saniculifolius* (Viviani) C. D. K. Cook
*Eucypris* cf. *crassa Müller*	*Ruppia drepanensis* Tineo ex Guss.
*Euplectella* sp.	*Salicornia europaea* Linné
*Eurytemora velox* Liljeborg	*Scirpus lacustris tabernaemontanii* (C.C.Gmel.) A.Löve & D.Löve
*Graeteriella* cf*. unisetigera* Graeter	*Scirpus litoralis* Schrad.
*Graeteriella* sp.	*Scirpus maritimus* Linné
*Halicyclops neglectus* Kiefer	*Spergularia heldreichii* Foucaud
*Ilyocypris monstrifica* Norman	*Tolypella hispanica* Nordstedt
*Ilyocypris* sp.	*Typha domingensis* Pers.
*Kurzia latissima* Kurz	*Veronica anagallis*-*aquatica* Linné
*Latona setifera* Müller	
*Leydigia acanthocercoides* Fischer	
*Leydigia leydigii* Leydig	
*Leydigia quadrangularis* Leydig	
*Leydigia* sp.	
*Macrothrix hirsuticornis* Norman & Brady	
*Macrothrix rosea* Liéven	
*Mesocyclops leuckarti leuckarti* Claus	
*Metacyclops gracilis* Liljeborg	
*Metacyclops planus* Gurney	
*Microcyclops* sp.	
*Microcyclops minutus* Claus	
*Microcyclops varicans* Sars	
*Moina macrocopa* Straus	
*Moina micrura* Baird	
*Moina* sp.	
*Oxyurella tenuicaudis* Sars	
*Paracyclops affinis* Sars	
*Pleuroxus aduncus* Jurine	
*Pleuroxus denticulatus* Birge	
*Pleuroxus* sp.	
*Pleuroxus striatus* Schödler	
*Pleuroxus trigonellus* Müller	
*Pleuroxus truncatus* Müller	
*Simocephalus expinosus* Koch	
*Simocephalus* sp.	
*Simocephalus vetulus* Müller	
*Tretocephala ambigua* Liljeborg	
*Triops cancriformis* Bosc	
*Tropocyclops prasinus* Fischer	
Unidentified *Limnocytheridae*	
*Vestalenula* sp.	

## Appendix 2: Detailed description of the statistical approaches used in this study

### Assessment of beta diversity and its nestedness and turnover components

In the beta diversity partitioning approach by Baselga and Orme (2012), beta diversity is expressed scientifically as the total Sørensen dissimilarity among ponds, to be separated into the components of dissimilarity due to species replacement (i.e., turnover) and dissimilarity due to nestedness. Here, I use this method to compute multiple-site dissimilarity measures that account for compositional species heterogeneity across ponds in the real examples and habitats in the perfect expression of turnover and nestedness for deriving diversity fractions. I calculated total Sørensen dissimilarity for invertebrates and macrophytes and their turnover and nestedness components. With this partitioning approach perfect turnover explains numerically the total dissimilarity or beta diversity (i.e., turnover = 1; nestedness = 0) while with perfect nestedness total beta diversity becomes 0 and nestedness and turnover are partitioned into equal fractions. In practice, this means that nestedness is responsible for the total dissimilarity. The analyses were carried out in the free statistical software R v. 2.15.1. (R Development Core Team 2012), using package *betapart* (Baselga and Orme 2012).

### Spatial modeling to assess structure in the geometries of the visuals

To reveal spatial structure in the visuals of beta diversity for each matrix (perfect nestedness, perfect turnover, invertebrates, macrophytes), I used a spatial modeling technique commonly used in ecology, which is capable of identifying spatial structure in study objects (Dray et al. 2006). This method is using a multivariate modeling technique based on Redundancy Analysis (RDA), which uses distance-based Moran Eigenvector Maps (dbMEM) to model space. Essentially, the dbMEM analysis produces a set of orthogonal spatial variables derived from the XY coordinates comprising the matrix. With data points arranged in a rectangular grid with equal distances to each other, like the pixels in the matrices created for this study (Figs. 1, 2), these dbMEM variables take the form of sine waves with distinct frequencies. The number of sine waves (or dbMEM variables) obtained depends on the number of points or pixels present in the matrix. These dbMEM variables are then used as explanatory variables in models of spatial relationships in the species matrices.

Next, a parsimonious spatial model for each matrix (perfect nestedness, perfect turnover, invertebrates, macrophytes) was produced by running a forward selection process on these dbMEM variables. This is done in the RDA analysis, which retains significant dbMEM variables. The RDA also linearly combines significant dbMEM variables that have been previously selected by the model, so that spatial patterns may be extracted from the matrices. To improve model performance and fulfill statistical assumptions of this modeling approach data matrices are generally transformed prior to the analyses, often concretely using the Hellinger-transformation (Legendre and Gallagher 2001).

In the next modeling step, species (pixels) or groups of species with similar spatial patterns are identified and collapsed onto independent RDA axes. The identified spatial patterns associated with each RDA axis are rigorously tested using permutations, so that the patterns identified are independent from each other. The resulting patterns can, but must not necessarily reflect hierarchical structures (that is, broad-scale vs fine-scale variation) in the matrices. That is, the technique is sensitive enough to identify even subtle differences in community structure at any spatial scale discernable given data resolution and extent, allowing for identification of independent patterns or orthogonal patterns in the matrices used for expressing beta diversity artistically.

The number of modeled spatial patterns of groups of pixels is deduced from the number of significant RDA axes, which synthesize the model information in spatial planes, and the ecological relevance of the spatial patterns or structure is quantified with the adjusted R^2^ values of the RDA axes. Finally, the overall spatial structure of a community is inferred from the number of significant axes in the RDA model. dbMEM analysis is powerful for detecting spatial patterns, but the method is inefficient in handling linear trends; therefore, the detrending of raw data is required prior to analysis (Dray et al. 2006). Although methods exist that account for linear trends (that is, asymmetric eigenvector maps; Blanchet et al. 2008), linearity is modeled according to explicit connectivity patterns among sites (for instance, upstream and downstream sites in a stream network) (Göthe et al. 2013). Because the pixels in the matrices used in this study have no specific connectivity patterns the dbMEM approach is suitable for identifying spatial relationships in these data. My initial analysis has revealed that no linear trend was present in the matrices; therefore, detrending was not required for the analyses presented here. All relevant analysis steps were carried out in R 2.15.1 (R Development Core Team 2012) with the packages *PCNM* (calculates dbMEM variables), *AEM* (Moran’s I spatial autocorrelation), *vegan* (Hellinger transformations, RDA), and *packfor* (forward selection). The analysis, calculation and reporting of statistics is fully automatic, based on a sequential execution of each modeling step in R.
